# Retroperitoneal solitary fibrous tumor of the pelvis with pollakiuria: a case report

**DOI:** 10.1186/1756-0500-5-593

**Published:** 2012-10-29

**Authors:** Takaaki Tsushimi, Takaharu Yagi, Naobumi Tomozawa, Hiromi Ohnishi

**Affiliations:** 1 Department of Surgery and Department of Pathology, Ehime Rosai Hospital, Minamikomatsubara-cho 13-27, Niihama, Ehime, 792-8550, Japan

**Keywords:** Pelvis, Solitary fibrous tumor, Pollakiuria

## Abstract

**Background:**

Solitary fibrous tumor (SFT) is rare soft tissue tumor, and it occurs most commonly in the pleura. Retroperitoneal SFT is generally found by palpable mass or abdominal distention. Here we report a case of SFT presenting pollakiuria.

**Case presentation:**

A 64-year-old man was referred to our hospital for pollakiuria. Contrasted-enhanced computed tomography revealed a heterogeneously enhanced pelvic tumor of approximately 10 × 8 × 7 cm. Invasion of the surrounding organs, distal metastasis, and lymph node swelling were absent. Therefore, surgical resection was performed. The resected specimen was a 13 × 8 × 5.5-cm encapsulated elastic hard tumor weighing 420 g. Histologically, the tumor consisted of oval or spindle cells growing in a random manner in a collagenous matrix. Immunohistochemically, the specimen was positive for CD34, bcl-2, as well as vimentin and negative for c-kit. On the basis of these findings, a retroperitoneal solitary fibrous tumor (SFT) of the pelvis was diagnosed.

**Conclusion:**

Surgery is the primary treatment for SFT, and pathologically negative margins are important for good prognosis.

## Background

Solitary fibrous tumor (SFT) is rare soft tissue tumor initially reported by Klemperer et al. [1]. It occurs most commonly in the pleura; however, extrathoracic SFT has also been reported [2-4]. Several cases of retroperitoneal soft tissue pelvic tumor have been reported [5]. SFT can be diagnosed preoperatively only by needle biopsy [6] because it is a rare lesion and not easily recognizable. In addition, no specific tumor markers have been identified or images published for this tumor type. We report a case of retroperitoneal SFT of the pelvis in a patient with pollakiuria. In this report, a review of related literature is also provided.

## Case presentation

**Figure 1:**
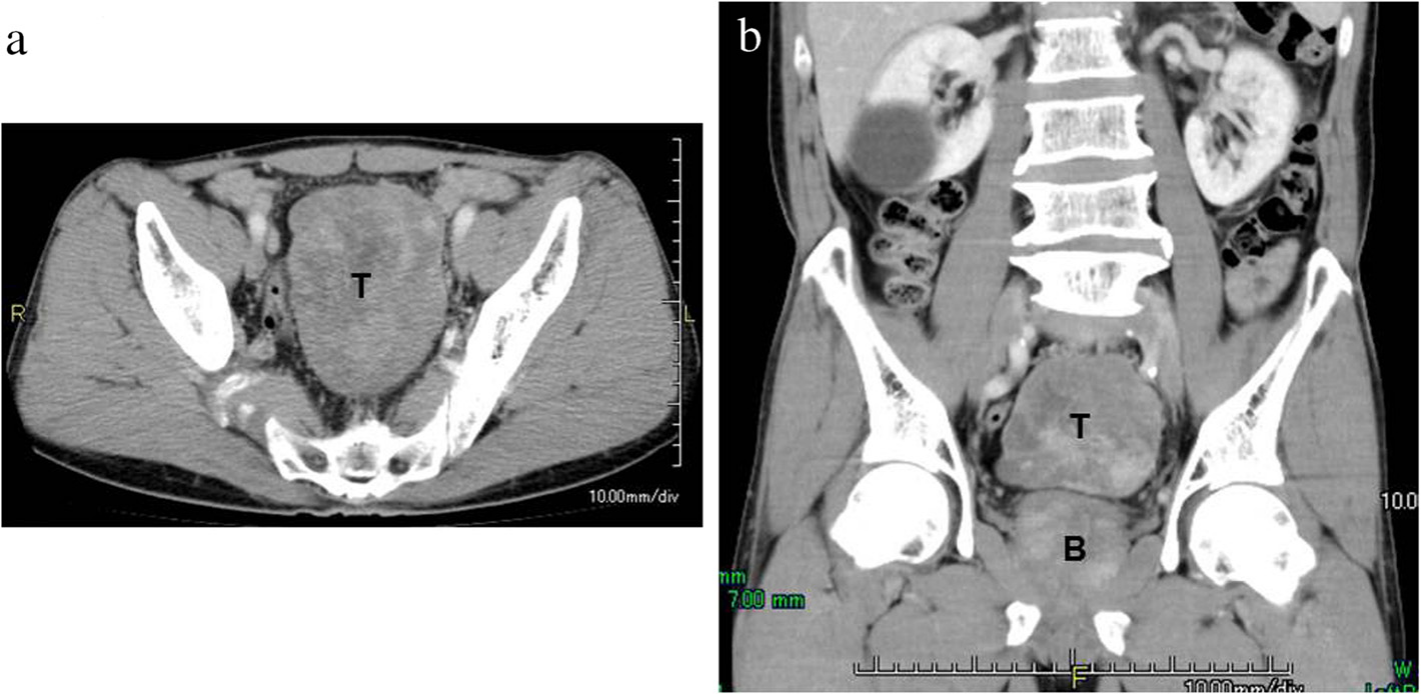
**CT showed a 10 × 8 × 7-cm, heterogeneously CE pelvic tumor that was slightly lobulated and included hypervascular regions.** (**a**) Transverse view. (**b**) Coronal view. T: tumor, B: bladder.

A 64-year-old man with a pacemaker for sick sinus syndrome complained of pollakiuria 5 months before presenting at our hospital. Results of abdominal examination were normal, and no palpable tumor or enlarged lymph node was found in the neck, abdomen, axilla, or inguinal regions. No abnormal findings were obtained from blood and urine examinations. Tests for carcinoembryonic antigen, alpha-fetoprotein, cancer antigen (CA) 19–9, CA125, and squamous cell carcinoma markers were all normal. Ultrasonography revealed a 10 × 8 × 6-cm low-echoic mass and a high-echoic region in the lower abdomen. Cystic regions were also observed. Contrasted-enhanced computed tomography (CE-CT) demonstrated a 10 × 8 × 7-cm moderately lobulated, internal, heterogeneous tumor with hypervascular regions; however, no calcification or fatty components were detected. Bladder compression by the tumor was observed. The tumor border was clear, and no evidence of direct invasion was detected (Figure 1a, b). CE-CT also revealed no liver or lung metastasis or intra-abdominal lymph node enlargement. Therefore, surgery was performed.

**Figure 2:**
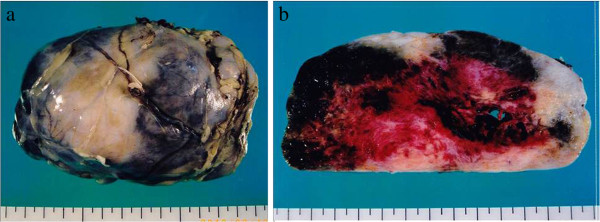
**Macroscopic findings.** (**a**) The resected specimen was 13 × 8 × 5.5 cm in size and 420 g in mass. The tumor was covered with a thick membrane and was elastic and hard. (**b**) A cross section revealed a solid tumor with a grayish-white cut surface, a cystic component, and a hemorrhagic focus.

**Figure 3:**
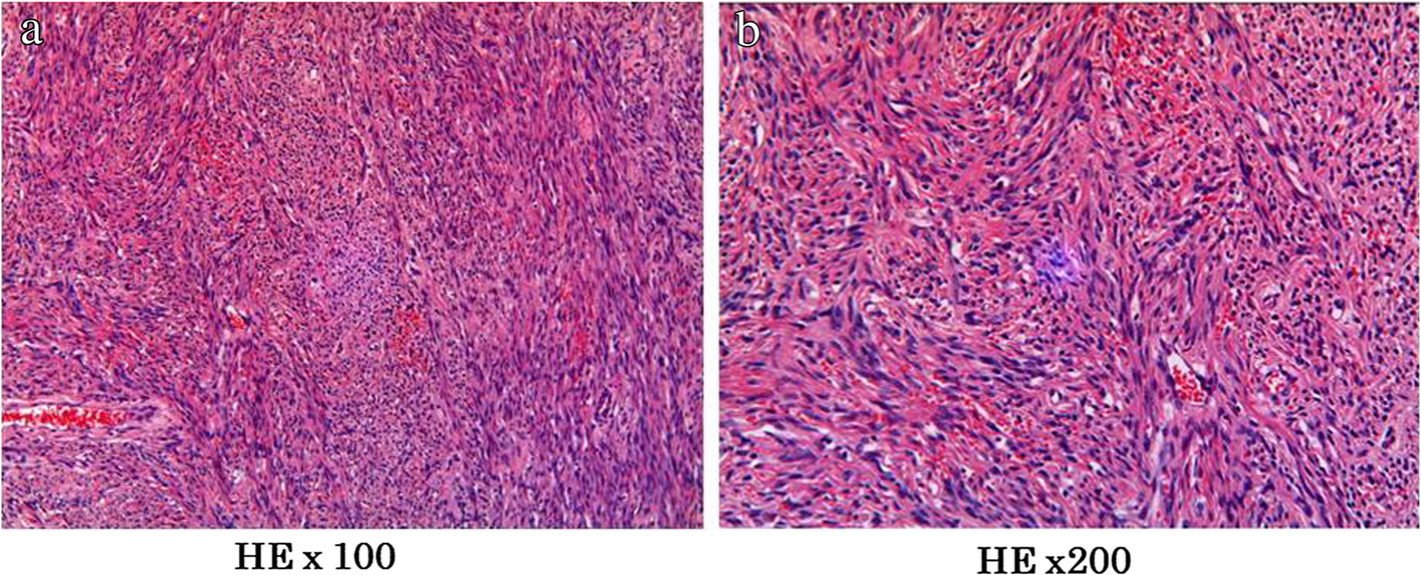
**Histopathological findings.** Oval or spindle cells in a random growth pattern with a collagenous matrix (hematoxylin–eosin staining). (**a**) × 100, (**b**) × 200.

**Figure 4:**
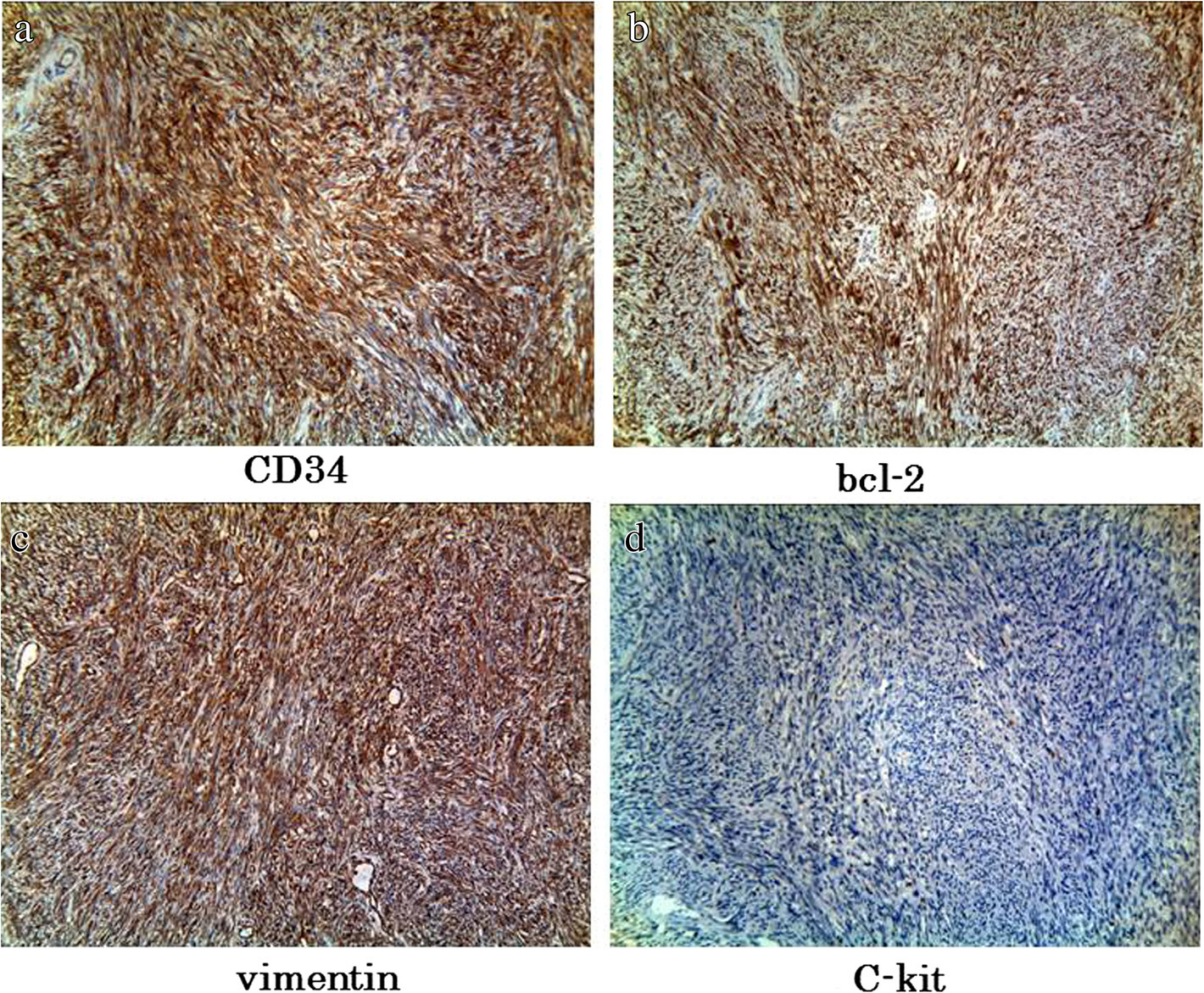
**Immunohistochemical findings: The specimen was positive for CD34, bcl-2, and vimentin, but negative for c-kit.** (**a**) CD34, × 100; (**b**) bcl-2, × 100; (**c**) Vimentin, × 100; (**d**) c-kit, × 100.

The tumor was found in the retroperitoneal pelvic area below the mesentery of the sigmoid colon. No intestinal adhesion or tumor invasion of the bladder or other organs was observed. Resection proceeded uneventfully. Operating time was 1 h 59 min, and intraoperative blood loss was found to be 241 ml. A 13 × 8.0 × 5.5-cm, 420-g, solid, hard elastic tumor covered with a thick fibrous membrane was excised. The cut surface was grayish-white and included a cystic component and hemorrhagic focus (Figure 2a, b). Pathologically, the tumor contained oval or spindle cells growing in a random pattern in a collagenous matrix (patternless pattern) (Figure 3a, b). Surgical margins were negative. Immunohistochemically, the specimen was positive for CD34, bcl-2, as well as vimentin, but negative for c-kit (Figure 4a–d). Immunohistochemical tests for p53, S-100, and alpha-smooth muscle actin were all negative. On the basis of these findings, retroperitoneal SFT of the pelvis was diagnosed. The postoperative course was uneventful, and pollakiuria immediately disappeared. Discharge occurred on postoperative day 10. No recurrence has been detected 20 months after surgery.

SFT is a soft tissue tumor first identified in the pleura by Klempler et al. in 1931 [1]. It is most common in the pleura, as demonstrated by Gold et al., who reported that 54 of 79 cases of SFT (68%) were found in the pleura [7]. SFT also arises in the retroperitoneal region [2], as in our case, and has been reported in the eye socket [3] and intracranial region [4]. Retroperitoneal SFT is usually diagnosed by the presence of an abdominal distention or a palpable mass [2]. Pollakiuria has been associated with SFT [8], as in our case, and it can also be related to difficulty with urination [9].

Pathological analysis of SFT showed poorly differentiated atypical tumor cells arranged in a random manner in hyalinized collagen fibers (patternless pattern) or in a hemangiopericytoma-like growth pattern [10]. Characteristic immunohistochemical findings of SFT include high positivity for CD34, which is known as the fibroblast-related antigen. Positive results of immunohistochemical testing for bcl-2 and vimentin and negative results for c-kit are also helpful in the diagnosis of SFT [10,11]. The tumor in this case demonstrated these characteristics of SFT.

The first-line treatment for SFT is surgical resection. Negative margins and complete surgical resection are essential for good prognosis [7]. Complete image inspection should be performed to determine the extent of tumor invasion around the organs as well as to ensure complete surgical resection and preservation of organ function. In this case, MRI was not possible because a pacemaker had been implanted previously. However, CE-CT revealed absence of tumor invasion of other organs, lymph node swelling and distal metastasis. Therefore complete resection was performed.

The World Health Organization classification of soft tissue tumor in 2002 categorized extrapleural SFT as a fibroblastic/myofibroblastic tumor of intermediate malignancy, which is defined as an ubiquitous mesenchymal tumor of possible fibroblastic type with a prominent hemangiopericytoma-like branching vascular pattern [10]. Pathological characteristics of malignant SFT include hypercellular lesions, cytological atypia, numerous mitoses (4 or more mitoses per 10 high-power fields), tumor necrosis, and/or infiltrating margins [7,10-12]. Vallat-Decouvelaere et al. reported malignancy in 8 of 92 cases of extrathoracic SFT, including local recurrence or distal metastasis, in 7 of which at least one of the typical pathological characteristics was observed in the primary tumor [12].

Various benign or malignant soft tissue tumors emerging from fat, muscle, and nerve may develop in the retroperitoneal region [5]. Some retroperitoneal tumors are diagnosed because of clinical symptoms or image inspections. However, a definite diagnosis is difficult to reach in many cases. Preoperative needle biopsy has been reportedly useful in diagnosis of SFT [6]. However, this technique carries the risk of peritoneal dissemination. Needle biopsy may be considered when complete surgical resection is not possible because some retroperitoneal tumors, including malignant lymphoma and malignant paraganglioma, may be sensitive to chemotherapy [5].

Takazawa et al. reported no recurrence in patients with SFT positive for both CD34 and bcl-2 [13]. Other researchers reported a correlation between expression of p53 and poor prognosis [6,14]. Yokoi et al. also found an association between expression of p53 in malignant SFT and poor prognosis, recurrence, nuclear atypia, high mitotic activity, and local invasion compared with that in benign SFT [14]. They suggested a possible association between p53 mutation and benign to malignant transformation in SFT. Therefore, expression of CD34, bcl-2, and p53 may be important in predicting the prognosis of patients with SFT.

## Conclusions

Complete surgical resection is the standard therapy for SFT and pathologically negative margins are important to achieve a good prognosis. Postoperative histochemical analysis revealed no CD34, bcl-2, or p53 expression after complete resection, and no atypical cells in the surgical margins, suggesting good prognosis in our case. However, careful follow-up is necessary, since recurrence has been reported more than 10 years after surgery in retroperitoneal SFT [15].

## Consent

Written informed consent was obtained from the patient for publication of this case report and any accompanying images. A copy of the written consent is available for review by the Editor-in-Chief of this journal.

## Competing interests

The authors declare that they have no competing interests.

## Authors’ contributions

TT analyzed and interpreted the patient data and drafted the manuscript. HO performed the histological examination of the tumor. NT and TY critically revised the manuscript. All authors read and approved the final manuscript.
